# SINGLE-CELL ANALYSIS OF SOMATIC MUTATIONS IN HUMAN LUNG REVEALS ASSOCIATION WITH TRANSCRIPTIONAL CHANGES IN AGING

**DOI:** 10.1093/geroni/igae098.1872

**Published:** 2024-12-31

**Authors:** Ruben De Man, Taylor Adams, John McDonough, Juan Cala-Garcia, Benjamin Moss, Xiting Yan, Ivan Rosas, Naftali Kaminski

**Affiliations:** Yale University School of Medicine, New Haven, Connecticut, United States; Yale University School of Medicine, New Haven, Connecticut, United States; McMaster University, Hamilton, Ontario, Canada; Baylor College of Medicine, Houston, Texas, United States; Baylor College of Medicine, Houston, Texas, United States; Yale University School of Medicine, New Haven, Connecticut, United States; Baylor College of Medicine, Houston, Texas, United States; Yale University School of Medicine, New Haven, Connecticut, United States

## Abstract

Age is a major risk factor for lung disease. Accumulation of somatic mutations has been implicated in both aging and cellular senescence. We analyzed somatic mutations called from single-cell RNAseq (scRNAseq) data. scRNAseq was performed on lung parenchyma samples from healthy donors (32 samples, 11-72 years, 21M/11F). Following standard pre-processing and cell type annotation, mutations were called using SComatic with the default parameters. Mutation burden was calculated for each cell type-sample combination by dividing the number of mutations by the number of callable sites. Our resulting scRNAseq dataset consisted of 199,400 cells, comprising 25 distinct cell types. Mutation burden was highest in alveolar type 1 (AT1) cells (93.6 mutations/MB), alveolar macrophages (79.1), and general capillary (gCap) cells (62.1). Mutation burden was positively correlated with age (r=0.28, p< 0.001). Globally, the top genes correlated with mutation burden included ubiquitin ligase genes (AMBRA1, ANAPC1, SEL1L, USP25, USP33) and DNA damage response genes (RAD50, PRKDC). Notably, mutation burden also correlated with expression of senescence marker CDKN2A (r=0.48, p< 0.05). In AT1 cells, mutation burden correlated with decreased expression of cell marker genes such as AGER (r=-0.64, p< 0.05) and HOPX (r=-0.83, p< 0.05). Similarly, gCap cells exhibited decreased expression of marker genes Il7R (r=-0.54) and VIPR1 (r=-0.64), while MAPK/ERK signaling genes were increased (p < 0.05). These genes were also significantly correlated with age in the same direction (p < 0.05). These results suggest that somatic mutation accumulation may contribute to age-associated transcriptional changes and loss of cell function, with cell types of the alveoli and endothelium experiencing the greatest effects.

